# Ancient connections among the European rivers and watersheds revealed from the evolutionary history of the genus *Telestes* (Actinopterygii; Cypriniformes)

**DOI:** 10.1371/journal.pone.0187366

**Published:** 2017-12-11

**Authors:** Ivana Buj, Zoran Marčić, Marko Ćaleta, Radek Šanda, Matthias F. Geiger, Jörg Freyhof, Annie Machordom, Jasna Vukić

**Affiliations:** 1 Division of Zoology, Department of Biology, Faculty of Science, University of Zagreb, Zagreb, Croatia; 2 Faculty of Teacher Education, University of Zagreb, Zagreb, Croatia; 3 National Museum, Prague, Czech Republic; 4 Leibnitz Institute of Freshwater Ecology and Inland Fisheries, Berlin, Germany; 5 Zoologisches Forschungsmuseum Alexander Koenig, Bonn, Germany; 6 Department of Biodiversity and Evolutionary Biology, Museo Nacional de Ciencias Naturales, Madrid, Spain; 7 Department of Ecology, Faculty of Science, Charles University, Prague, Czech Republic; National Cheng Kung University, TAIWAN

## Abstract

In order to better understand the complex geologic history of the Mediterranean area, we have analysed evolutionary history, phylogeographic structure and molecular diversity of freshwater fishes belonging to the genus *Telestes*. As primary freshwater fishes distributed largely in the Mediterranean basin, this genus represents a suitable model system for investigating the historical biogeography of freshwater drainage systems in southern Europe. In this investigation we have included samples representing all *Telestes* species and based our analyses on one mitochondrial and one nuclear gene. We have investigated phylogenetic structure inside the genus *Telestes*, estimated divergence times, reconstructed ancestral distribution ranges and described intraspecific molecular diversity. Diversification of *Telestes* started in the Early Miocene, when the ancestors of *T*. *souffia*, lineage comprising *T*. *croaticus* and *T*. *fontinalis*, and the one comprising *T*. *pleurobipunctatus* and *T*. *beoticus* got isolated. The remaining species are genetically more closely related and form a common cluster in the recovered phylogenetic trees. Complex geological history of southern Europe, including formation of continental bridges, fragmentation of landmass, closing of the sea corridor, local tectonic activities, led to complicated biogeographical pattern of this genus, caused by multiple colonization events and passovers between ancient rivers and water basins. Especially pronounced diversity of *Telestes* found in the Adriatic watershed in Croatia and Bosnia and Herzegovina is a consequence of a triple colonization of this area by different lineages, which led to an existence of genetically distinct species in neighboring areas. Significant intraspecific structuring is present in *T*. *souffia*, *T*. *muticellus*, *T*. *croaticus* and *T*. *pleurobipunctatus*. Besides in well-structured species, elevated levels of genetic polymorphism were found inside *T*. *turskyi* and *T*. *ukliva*, as a consequence of their old origin and unconstrained evolutionary history.

## Introduction

Traces of the evolutionary history of animals, saved in their genetic composition and polymorphism, are very useful for revealing the biogeographic history inside their distribution ranges, including type and time frame of past geologic events. In regions where geological history was especially complex, and geological evidences are often scarce or ambiguous, revelation of evolutionary history of animal taxa is even more important in understanding the complicated past of geographic regions. This is especially true for freshwater fishes, due to their inevitable connection to freshwater systems and their geologic history.

**Fig 1:**
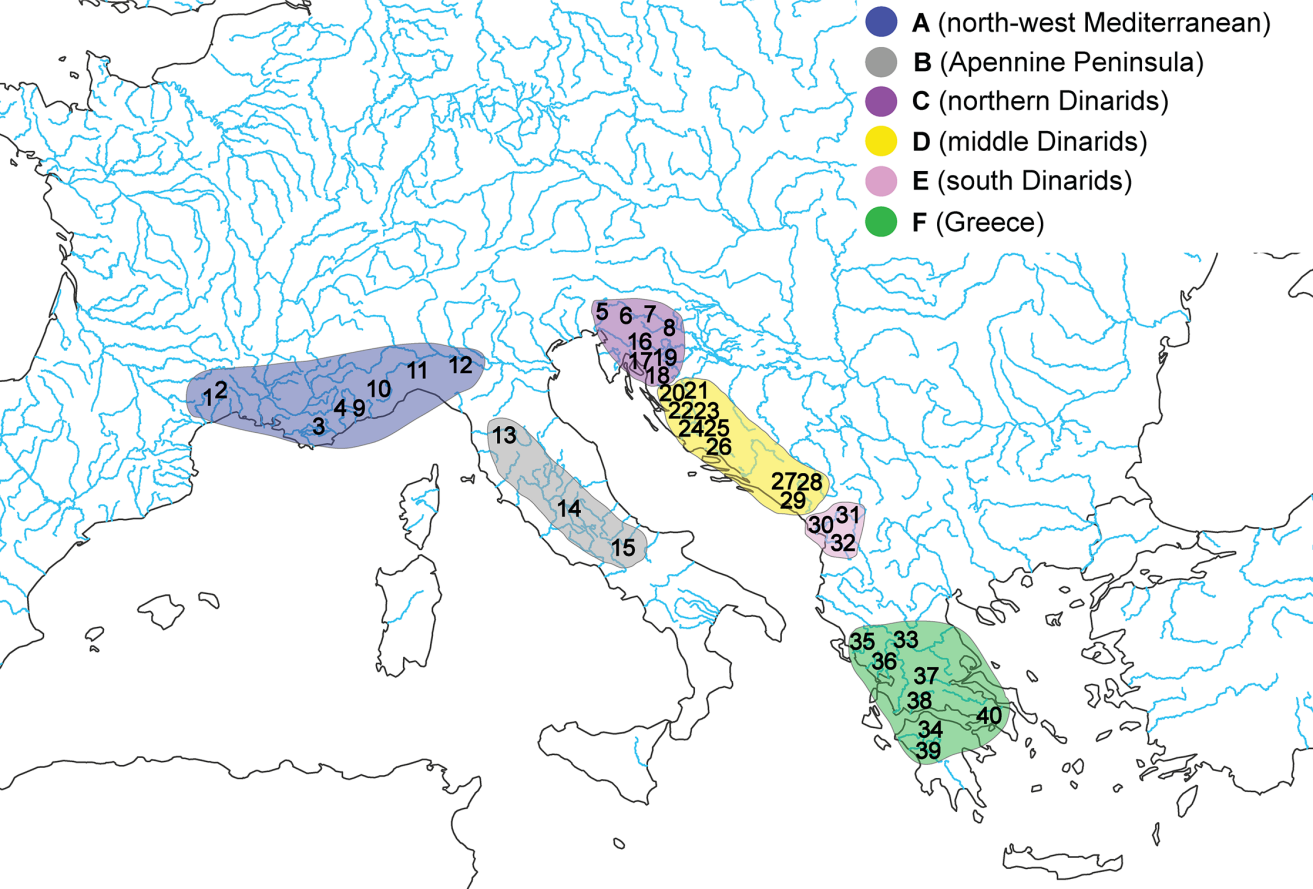
Map of the investigated area with marked sampling localities. 1- Lergue, 2-Hérault, 3-Argens, 4-Var, 5-Soča, 6-Gradaščica, 7-Jevnica, 8-Bregana, 9-Roya, 10-Bevera, 11-Staffora, 12-Po, 13-Arno, 14-Tiber, 15-Volturno, 16-Jasenak field, 17-Sušik, 18-Studenac, 19-Šmit lake, 20-Jadova, 21-Ričica, 22-Suvaja, 23-Krbava, 24-Čikola, 25-Vinelić, 26-Cetina, 27-Gatačko field, 28-Dabarsko field, 29-Konavle, 30-Zeta, 31-Morača, 32-Cijevna, 33-Kalamas, 34-Pinios, 35-Pavllo, 36-Louros, 37-Acheloos, 38-Evinos, 39-Alfios, 40-Kifissos. Different colors represent ranges as used for S-DIVA analysis.

The genus *Telestes* Bonaparte, 1837 comprises primary freshwater fishes that are obligate inhabitants of moderately cold waters of riverine ecosystems. Because their current distribution has not been significantly influenced by stocking due to a lack of commercial relevance, it most likely still reflects the genus’ biogeographic history [1]. Therefore, this genus represents a suitable model for investigating the historical biogeography of European freshwater drainage systems. *Telestes* belongs to the Leuciscinae subfamily and contains 14 species, mainly distributed in the rivers of the Mediterranean basin (Fig 1): *T*. *beoticus* (Stephanidis, 1939) distributed in the Kiffisos and Assopos drainages in Greece; *T*. *croaticus* (Steindachner, 1866) inhabiting the Jadova and Ričica Rivers in Croatia; *T*. *dabar* Bogutskaya, Zupančič, Bogut & Naseka, 2012 found in the Dabarsko karstic field in Bosnia and Herzegovina; *T*. *metohiensis* (Steindachner, 1901) distributed in the Nevesinjsko, Gatačko and Cernica karstic fields in Bosnia and Herzegovina; *T*. *miloradi* Bogutskaya, Zupančič, Bogut & Naseka, 2012 restricted to the Konavosko karstic field in Croatia; *T*. *montenigrinus* (Vuković, 1963) inhabiting the lake Skadar basin in Montenegro and Albania; *T*. *muticellus* (Bonaparte, 1837) being widely distributed in Italy and southern Switzerland; *T*. *pleurobipunctatus* (Stephanidis, 1939) distributed in southern Albania and Greece; *T*. *turskyi* (Heckel, 1843) from the Čikola River in Croatia; and *T*. *ukliva* (Heckel, 1843) restricted to the Cetina River drainage in Croatia. Four species are found in the Black Sea basin: *T*. *karsticus* Marčić & Mrakovčić, 2011 inhabiting few small streams in the mountain region in Croatia; *T*. *polylepis* (Steindachner, 1866) from the Šmit Lake in Croatia; *T*. *fontinalis* (Karaman, 1972) in Krbavsko karstic field; and *T*. *souffia* (Risso, 1827) with a broader distribution range comprising rivers belonging to both Mediterranean (from Aude to Var drainages in France and Switzerland, Soča drainage in Slovenia and Italy, Rhine drainage in Germany and Switzerland) and Black Sea drainages, where it is widespread in headwaters of the Danube drainage from Germany downriver to Romania. The distribution ranges of *T*. *karsticus*, *T*. *polylepis* and *T*. *fontinalis* are situated on the border line between the Adriatic and the Black Sea basin and these rivers only have subterranean connections to the Black Sea basin.

Despite several attempts to estimate timing of divergence events inside the genus *Telestes*, the results were far from unambiguous, most likely as a consequence of different molecular clock calibrations, but also due to discrepancies in species sets investigated. In fact, none of the previous studies included all *Telestes* species. [2] estimated the separation of *Telestes* from other leuciscinids to around 14.3 MYA (million years ago), while intrageneric divergences presumably started to occur around 12.5 MYA, which is the time frame of the onset of Dinarid orogenesis. [3], on the other hand, estimated diversification within *Telestes* to around 7.8 MYA. [1] concluded that *T*. *polylepis*, *T*. *turskyi*, *T*. *croaticus* and *T*. *metohiensis* separated from *T*. *souffia* 5.6–7.9 MYA. [4] estimated the separation of four *Telestes* species (*T*. *souffia*, *T*. *muticellus*, *T*. *montenigrinus* and *T*. *pleurobipunctatus*) to have occurred 6.7–4.9 MYA, however, their molecular clock calibration was based on the opening of the Corinthian Gulf. New data reveal that a Corinthian Gulf opening was not a vicariant event for this genus, and therefore is unsuitable for molecular clock calibration. [5] calibrated their molecular clock using a mutation rate of 1% and 2% substitutions per million years and estimated separation of *T*. *souffia* and *T*. *muticellus* to around 5.3 MYA. Based on [4] and [5] the divergences inside *T*. *souffia* occurred 0.5–1 MYA. Generally it is considered that speciation within the genus *Telestes* was based on allopatric isolation [4]. Hybrids of *T*. *muticellus* and *T*. *souffia* have only been reported from the margins of their adjacent ranges [5], [3], [4].

In depth genetic analyses have been conducted and the demographic history partially revealed for some *T*. *muticellus* populations [6], [3], [7], [8] and *T*. *souffia* [9], [5], [4], without identifying clearly the geological events behind the phylogenetic patterns observed.

The aim of this research was to reveal the importance of different geological events on the evolutionary history of the genus *Telestes*, but also to uncover possible local events and circumstances that shaped the recent distribution and diversity of the species involved. Moreover, our goal was to document differences in present genetic structure and polymorphisms among species that reflect their demographic history, which could help unraveling the complex geological past of the Mediterranean area.

## Materials and methods

### Ethics statement

This investigation was conducted entirely in accordance with ethical standards and Croatian legislation. The Ethical Committee of the Faculty of Science, University of Zagreb, approved the work. Permission for sampling was issued by competent authorities: for sampling on localities within Croatia (Jadova, Suvaja, Ričica, Krbava, Čikola, Cetina, Vinelić, Studenac, Sušik, Jasenak field, Šmit lake, Gradaščica, Bregana and Konavosko field) permit was issued by the Ministry of Nature Protection; for Greek localities (Pavllo, Acheloos, Louros, Pinios, Evinos, Kalamas, Alfios, Kifissos) by the Greek Ministry of Environment, Energy and Climate Change; for sampling on localities in Montenegro (Cijevna, Morača, Zeta) permission was issued by Ministry of Agriculture and Rural Development; Italian samples (localities Bevera, Staffora, Arno, Tiber, Volturno, Po) were obtained based on permissions from following authorities: ‘Amministrazione Provinciale di Cuneo–Servizio Caccia e Pesca’, ‘Citta Metopolitana di Torino–Servizio Tutela Fauna e Flora’, ‘Parco del Monviso’, ‘Ente di gestione delle Aree Protette del Po e della collina Torinese’, ‘Ente Tutela Pesca del Friuli Venezia Giulia’, ‘Administrazione Provinciale di Arezzo–Servizio Caccia e Pesca’; on French localities (Argens, Herault, Lergue and Var) permission from ‘Conseil Superieur de la Peche’ was obtained; and for localities within Bosnia and Herzegovina permit was issued by ‘Republički zavod za zaštitu kulturno-istorijskog i prirodnog nasljeđa, Banja Luka’.

We have collected samples from 40 localities covering the whole geographic range of the genus *Telestes* (Fig 1, Table 1) and representing all 14 recognized species. For species presented with more than one population, namely those distributed on more than one locality that are not connected with permanent ground connections, samples were obtained from more than one locality. Sampling was conducted by electrofishing. Small piece of fin tissue was taken from each individual and deposited in ethanol or frozen for further analyses. Two molecular markers were employed in this investigation: the mitochondrial gene for cytochrome *b* (cyt *b*) and the nuclear RAG1 gene with altogether 2490 base pairs (bp) analyzed. The cyt *b* gene is considered as the most useful marker in recovering phylogenetic relationships among closely related taxa [10], [11] and its suitability for taxonomic and population genetic studies was proven in several previous investigations on various vertebrates (e.g. [10], [12], [13]). RAG1 gene, on the other hand, due to its lower mutation rate, is adequate for phylogenetic investigation on higher level, namely discovering relationships among more distantly related species. Inclusion of both, maternally inherited and biparentally inherited markers shall enable investigation of phylogenetic structure and evolutionary history of this genus, but also point out to eventual hybridization.

**Table 1:** Sampling localities and number of specimens belonging to certain species collected at each locality.

Species	Locality	Basin (country code)	cyt b sequences	RAG1 sequences	GenBank accession no.
***T*. *croaticus***	Jadova	Adriatic (CRO)	13	8	MG372513-28, MG372665-71
	Suvaja	Adriatic (CRO)	5	2	
	Ričica	Adriatic (CRO)	4	4	
	TOTAL		**22**	**14**	
***T*. *fontinalis***	Krbava	Danube (CRO)	**13**	**12**	MG372532-38, MG372672-74
***T*. *turskyi***	Čikola	Adriatic (CRO)	**8**	**4**	MG372552-58, MG372680
***T*. *ukliva***	Cetina	Adriatic (CRO)	9	4	MG372539-47, MG372587-90, MG372678-79
	Vinelić	Adriatic (CRO)	5		
	TOTAL		**14**	**4**	
***T*. *karsticus***	Studenac	Danube (CRO)	10	12	MG372509-12, MG372529-31, MG372675-76
	Sušik	Danube (CRO)	13	14	
	Jasenak field	Danube (CRO)	6	2	
	TOTAL		**29**	**28**	
***T*. *polylepis***	Šmit lake	Danube (CRO)	**5**	**6**	JN188377-80, MG372677
***T*. *montenigrinus***	Cijevna	Adriatic (MNE)	5	4	MG372559-67, MG372622, MG372681-83
	Morača	Adriatic (MNE)	7	2	
	Zeta	Adriatic (MNE)	14	10	
	TOTAL		**26**	**16**	
***T*. *pleurobipunctatus***	Pavllo	Ionian (GRE)	6	4	MG372568-73, MG372623-55, MG372684-95
	Acheloos	Ionian (GRE)	6	10	
	Louros	Ionian (GRE)	15	22	
	Pinios	Ionian (GRE)	8	14	
	Evinos	Ionian (GRE)	10	10	
	Kalamas	Ionian (GRE)	10	18	
	Alfios	Ionian (GRE)	10	12	
	TOTAL		**65**	**90**	
***T*. *beoticus***	Kifissos	Aegean (GRE)	**11**	**14**	MG372658-64, MG372725-26
***T*. *muticellus***	Bevera	Ligurian (ITA)	6	12	MG372574-77, MG372591-621, MG37696-711
	Staffora	Adriatic (ITA)	6	2	
	Arno	Tyrrhenian (ITA)	9	10	
	Tiber	Tyrrhenian (ITA)	12	12	
	Volturno	Tyrrhenian (ITA)	10	12	
	Po	Adriatic (ITA)	11	16	
	Roya	Ligurian (ITA)	1		
	TOTAL		**55**	**64**	
***T*. *souffia***	Gradaščica	Danube (CRO)	9	12	MG372548-51, MG372578-86, MG372656, MG372712-22
	Bregana	Danube (CRO)	5	2	
	Jevnica	Danube (SLO)	6	4	
	Soča	Adriatic (SLO)	5	2	
	Argens	Mediterranean (FRA)	6	6	
	Herault	Mediterranean (FRA)	6	6	
	Lergue	Mediterranean (FRA)	6	4	
	Var	Mediterranean (FRA)	6	4	
	TOTAL		**49**	**40**	
***T*. *metohiensis***	Gatačko field	Adriatic (BIH)	**6**	**8**	MG372503-08, MG372723-24
***T*. *dabar***	Dabarsko field	Adriatic (BIH)	**11**	**4**	MG372496-502
***T*. *miloradi***	Konavosko field	Adriatic (CRO)	**5**		MG372657

Country codes: CRO-Croatia, MNE-Montenegro, GRE-Greece, ITA-Italy, FRA-France, BIH-Bosnia and Herzegovina.

Total genomic DNA was extracted from fin tissue samples using a standard extraction product (DNeasy tissue kit, Qiagen). Polymerase chain reaction (PCR) amplifications were performed using HotStarTaq Master Mix (Qiagen) and primers GluF and ThrR [14] for cyt *b* gene, and RAG1F1 and RAG1R1 [15] for RAG1. Amplification of cyt *b* gene was conducted through 35 PCR cycles with 45 s of denaturation at 92°C, 90 s of annealing at 48°C and 1 min and 45 s of extension at 72°C. PCR protocol for RAG1 gene comprised 5 cycles with 40 s at 94°C, 1 min at 60°C, 2 min at 72°C, followed by 35 cycles with 30 s at 95°C, 1 min at 56°C and 2 min at 72°C. Sequencing was carried out by Macrogen Service Centre (Amsterdam, Nederland) with internal primer pairs CB4-GLU (5’CCTGAAAYATYGGYGTRGT3’) and PHOX-THR (5’AGGAGGAARTGRAATGCGAA3’) (Doadrio and Perea, personal communication) for cyt *b*, and RAG3F (5’GGGTGTGTCAGYGAGAAGCA3’) (Quenouille et al. 2004 in [2]) and RAG6R (5’ATGGCTTTCCGCTCTGCTAC3’) (Doadrio and Perea, personal communication) for RAG1. We have obtained cyt *b* sequences from 319 samples and RAG1 sequences from 152 samples.

Homologous regions of both genes were aligned manually against previously published sequences. Chromatograms and alignments were checked visually and were found to contain no gaps or stop codons. In order to determine nuclear haplotypes in heterozygous individuals, Bayesian statistical method implemented in PHASE 2.1 software [16], [17] was conducted. The analysis was run five times with different values of the seed of the pseudo-random number generator. Each run consisted of a burn-in-period of 100 followed by 1000 iterations. In order to test whether all mutations were selectively neutral, statistical tests D* and F* [18] and Tajima’s test [19] were conducted using DnaSP v5 [20]. The same software was employed to estimate the recombination parameter, R [21], and the minimum number of recombination events, RM [22], for nuclear data set.

Phylogenetic reconstruction was conducted with the aim of revealing the phylogenetic structure inside the genus *Telestes*, i.e. confirming the position of already investigated *Telestes* populations and revealing phylogenetic relationships for populations analysed for the first time. It was based on two methods of phylogenetic inference: maximum parsimony (MP), implemented in PAUP (version 4.0b10; [23]), and Bayesian inference (BAY), implemented in MrBayes (version 3.1.2; [24]). For MP analysis, a heuristic search mode was used, with randomized input orders of taxa, and TBR branch swapping with all codon sites and nucleotide substitutions types weighted equally. Nonparametric bootstrapping (1000 pseudo-replicates, 10 additional sequence replicates) was used to assess branch support (BS). Each BAY analysis consisted of two simultaneous runs. For each, Markov Chain Monte Carlo was run four times for three million generations with trees sampled every 100 generations. The first 20% of the sampled trees were discarded and Bayesian posterior probabilities (BPP) were estimated from the 50% majority-rule consensus tree of the retained trees. For rooting of cyt *b* phylogenetic tree we used a sequence of *Squalius cephalus*, more distant representative of the same subfamiliy [2], and of *Cyprinus carpio*, that belongs to the same family, but to a different subfamily. A sequence of *Squalius squalus* was used for rooting the RAG1 phylogeny. As the most suitable model of sequence evolution, TN93 model was identified using MEGA6 software and used for BAY, as well as *BEAST analyses (see later).

Additional method, median-joining approach (MJ) using Network 4.5.1.6. software (Fluxus Technology Ltd.) was employed on RAG1 data set. In a resulting phylogenetic network it is possible to notice horizontal gene transfers, so this approach is especially adequate for phylogenetic reconstruction based on nuclear genes.

Divergence times between investigated species, as well as between phylogenetic lineages, were estimated by a Bayesian MCMC coalescent method, using BEAST 2.4.7software [25]. The analysis was conducted on cyt *b* data set and on the concatenated data set (cyt *b* and RAG1). Concatenation was achieved using Mesquite 2 software [26]. The rate homogeneity across phylogenetic lineages was assessed by the log-likelihood ratio test (LRT), comparing the likelihood of phylogenetic trees (reconstructed using maximum likelihood approach) with and without molecular clock enforcement in PAUP*. Since the likelihood scores were the same in both cases for cyt *b* data set, we applied a strict molecular clock. Since we aimed to reconstruct species tree and gene tree at the same time, while preparing an input file for analysis we used StarBeast template in BEAUti software. This analysis is based on a multispecies coalescent model, which allows inclusion of an algorithm of coalescence in the speciation process. The number of MCMC steps (the length of chain) was twenty millions. The molecular clock calibration was conducted based on the divergence rate of cyt *b* gene of 0.4% per lineage per million years [2]. For concatenated data set we employed relaxed molecular clock and divergence rate of 0.2% per lineage per MYA, based on [2].

Ancestral distribution ranges of lineages were reconstructed using Statistical Dispersal-Vicariance Analysis implemented in S-DIVA software [27]. This method reconstructs the ancestral distribution in a phylogeny by optimizing a three-dimensional cost matrix, in which extinctions and dispersals ‘cost’ more than vicariance, and it determines statistical support for ancestral range reconstruction [27]. Reconstruction of ancestral geographic ranges was conducted based on the concatenated data set in order to investigate bi-parental origins. An input set of trees was obtained by BAY analysis. Altogether six recent geographic ranges for *Telestes* were denoted (marked in the Fig 1).

Pairwise comparisons of uncorrected sequence divergence (p-distances) of both genes were analysed using MEGA version 3.1 [28].

The level of intraspecific genetic diversity was estimated by calculating several measures of DNA polymorphism for each genetic marker, employing DnaSP v.5: number of polymorphic sites (S), haplotype diversity (Hd), average number of nucleotide differences (K) and nucleotide diversity (π). Furthermore, the frequency of each haplotype was calculated as percentage of a certain haplotype in a population. In the intraspecific genetic diversity estimation we did not include sequences of introgressed individuals, in order not to overestimate measures; all measures are based on sequences belonging to a single species, as recognized by current taxonomy.

## Results

Analyses of phylogeny and population genetics were based on 319 cyt *b* sequences that were 1140 base pairs (bp) long and 304 RAG1 phased alleles with length of 1350 bp. Sequences of both genes were obtained for all species, with the exception of *T*. *miloradi*, in which only cyt *b* gene was successfully amplified. Within the cyt *b* data set 173 unique haplotypes were found, while the RAG1 gene revealed 62 haplotypes. The proportion of heterozygous individuals in the overall sample was 41.5%. Based on the neutrality tests we can conclude that both investigated genes are in mutation-drift equilibrium. Fu & Li’s D* and F* statistics, as well as Tajima’s D were not statistically significant (p>0.05) for RAG1 in all species and for cyt *b* in the majority of them (with the exception of all statistics for *T*. *karsticus* and Tajima’s D in *T*. *beoticus*). The minimum number of recombination events was much smaller than the number of mutations in RAG1 (6 vs. 92), implying that recombination did not affect the phylogenetic pattern.

The maximum parsimony tree based on cyt *b* has a length of 1308, consistency index (CI) 0.4549, homoplasy index (HI) 0.5451 and retention index (RI) 0.9340. For the RAG1 MP tree those measures are: length = 159, CI = 0.6478, HI = 0.3522, RI = 0.8261. Out of 1140 sites in cyt *b* 438 (38%) were polymorphic and 326 (28.6%) parsimony informative. In RAG1 sequences, there were 87 (6.4%) polymorphic sites, out of which 68 (5% of total sites) were parsimony informative. As expected, the number of parsimony informative characters was much lower in the nuclear gene, leading to a lower resolution of the RAG1 phylogeny.

**Fig 2:**
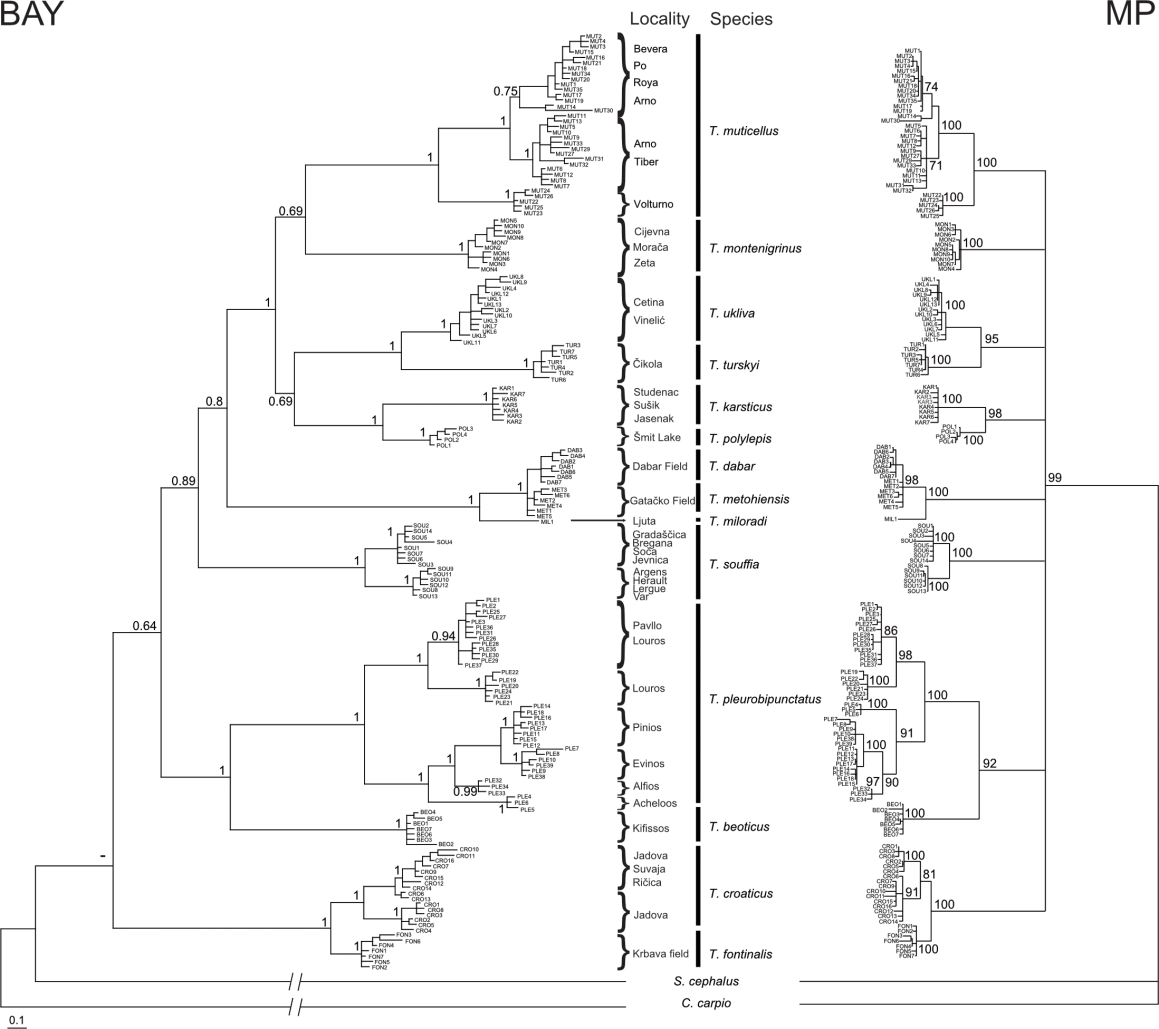
Phylogenetic tree of cyt *b* sequences based on Bayesian (BAY) and Maximum parsimony (MP) inferences. Numbers at nodes represent Bayesian posterior probabilities and MP branch support.

Even though the two methods of phylogenetic inference employed on the cyt *b* data set did not yield trees with exactly the same topologies, the internal structure of the genus is similar in both (Fig 2). Species recognized by current taxonomy are presented with separate evolutionary sublineages (for purposes of this investigation defined as the smallest monophyletic groups containing tips in a phylogram) or lineages (monophyletic units that are further divided into sublineages) in both phylogenetic trees. The only exceptions are *T*. *dabar* and *T*. *metohiensis* that form a single sublineage. On the other hand, intraspecific structuring of three widespread species (*T*. *pleurobipunctatus*, *T*. *muticellus*, *T*. *souffia*) and one endemic species (*T*. *croaticus*) is pronounced; each of them consists of more than one sublineage. Compared to MP, the BAY analysis delivered a better resolution of the interspecific relationships (MP resulted in a soft polytomy of all *Telestes* lineages). Both phylogenies support a sister position of *T*. *croaticus* and *T*. *fontinalis*; *T*. *beoticus* and *T*. *pleurobipunctatus*; *T*. *turskyi* and *T*. *ukliva*; *T*. *polylepis* and *T*. *karsticus*; as well as *T*. *miloradi* and *T*. *metohiensis*/*T*. *dabar*. A similar result as BAY analysis was also obtained with the BEAST analyses (presented later), where the Greek *Telestes* species (*T*. *pleurobipunctatus* and *T*. *beoticus*) separate very early from the main *Telestes* lineage, and *T*. *fontinalis*/*T*. *croaticus* lineage cluster in a basal position.

**Fig 3:**
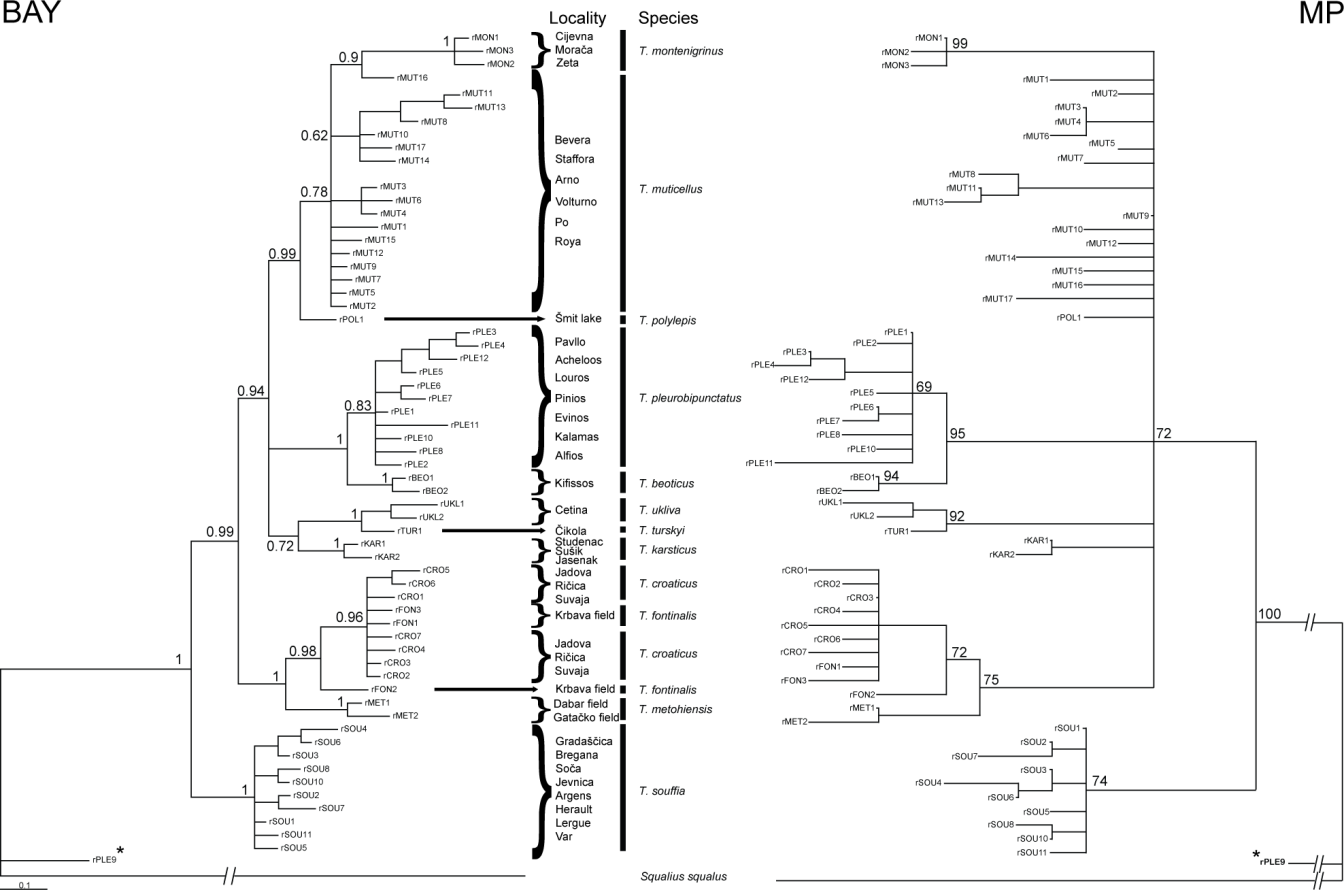
Phylogenetic tree of RAG1 sequences based on Bayesian (BAY) and Maximum parsimony (MP) inferences. Numbers at nodes represent Bayesian posterior probabilities and MP branch support. Asterisk denotes one allele from the TEEV7 sample, which belongs to *Squalius* sp.

The overall topology of the RAG1 phylogenies with the two different methods is similar (Fig 3), but shows some differences to cyt *b* phylogenetic trees. Those differences regard mainly intraspecific structuring, whereas separation of the majority of species can also be seen in the RAG1 phylogenies (with the exception of *T*. *dabar* and *T*. *metohiensis* that share the same nuclear haplotypes; but also *T*. *croaticus* and *T*. *fontinalis*, whose haplotypes are distinct, but clustered together). As in the analysis of the cyt *b* data, better resolution was achieved by the Bayesian inference, while maximum parsimony resulted in several soft polytomies. Both methods of phylogenetic inference based on the nuclear marker separated *T*. *souffia* from the cluster containing the remaining *Telestes* species and showed closer relatedness of *T*. *metohiensis* with *T*. *croaticus* and *T*. *fontinalis*, which was not the case in cyt *b* phylogenies. The remaining species (*T*. *karsticus*, *T*. *polylepis*, *T*. *turskyi*, *T*. *ukliva*, *T*. *beoticus*, *T*. *pleurobipunctatus*, *T*. *muticellus* and *T*. *montenigrinus*) form a common cluster in RAG1 phylogenies, but with lesser resolved inter- and intraspecific structuring, especially in MP phylogenetic tree.

**Fig 4:**
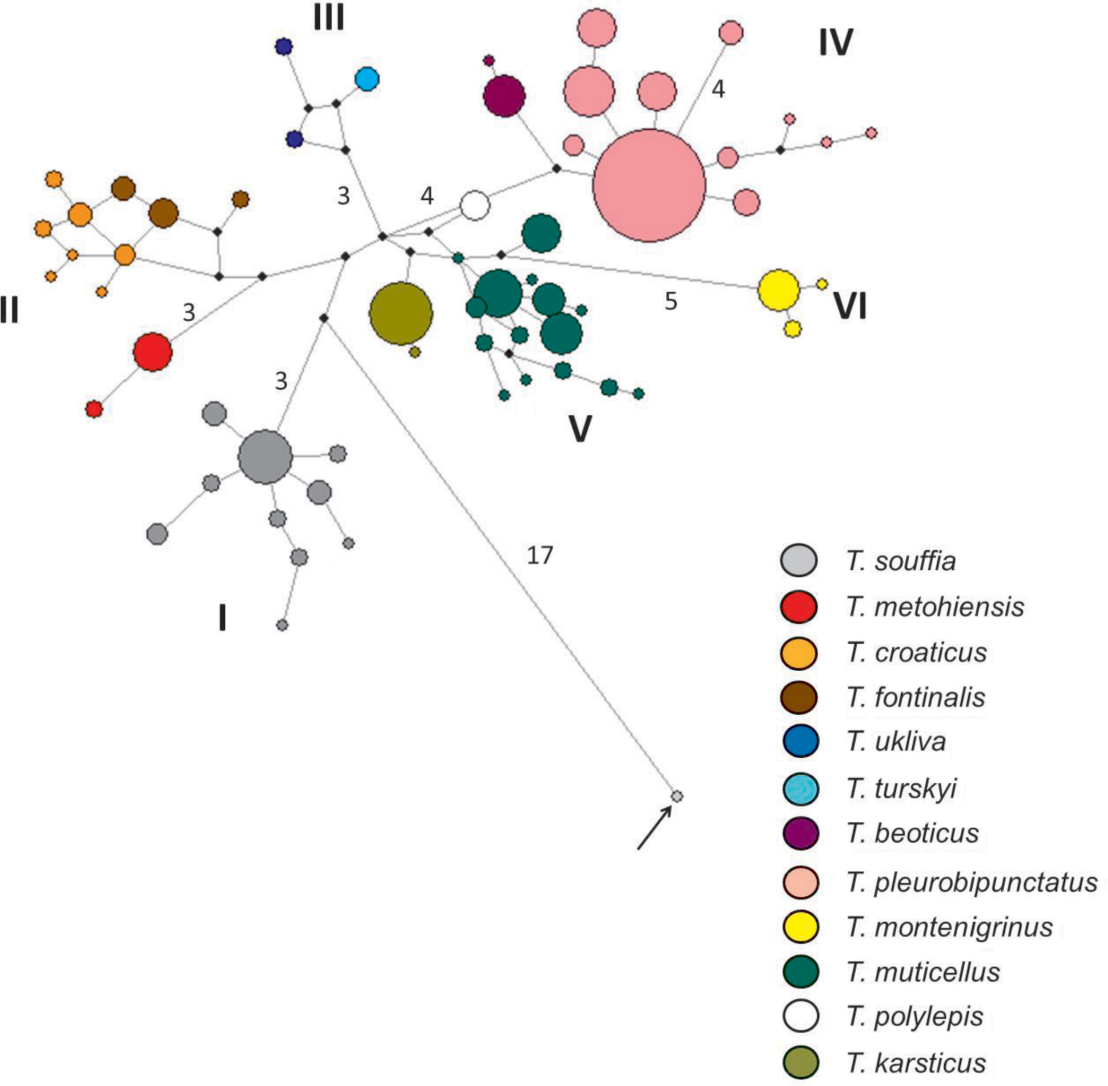
Median-joining network of nuclear haplotypes. Black circles represent median vectors. The number of mutational steps is displayed by the branches if higher than 2. Haplotype clusters are marked with roman numbers and haplotypes belonging to different species are presented with different colors. The arrow marks one allele from the TEEV7 sample, which belongs to *Squalius* sp.

MJ network of RAG1 haplotypes gave a similar, but clearer picture of the intrageneric structure of *Telestes* (Fig 4). All haplotypes are grouped in six distinct units: group I comprises only *T*. *souffia*; group II consists of *T*. *fontinalis*, *T*. *croaticus* and *T*. *metohiensis*, that is distinct from the first two species; sequences of *T*. *ukliva* and *T*. *turskyi* form group III; group IV contains two Greek species: *T*. *beoticus* and *T*. *pleurobipunctatus*; whereas group V comprises *T*. *muticellus*, *T*. *polylepis* and *T*. *karsticus*. Even though *T*. *montenigrinus* (group VI) seems to originate from an ancestor belonging to group V, it is, nevertheless, distinct from the remaining species in this cluster, separated by at least five mutational steps. Similarly to the RAG1 phylogenetic trees, groups III-VI assemble into a single cluster. One sequence found in a heterozygous *T*. *pleurobipunctatus* (rPLE9) is very distinct from all *Telestes* species (by at least 20 mutations), just as it was in the RAG1 phylogenetic tree.

Two samples from the French Bevera River, at the distribution border between *T*. *souffia* and *T*. *muticellus* possess genetic material of two species: sample TEBE5 shows one nuclear haplotype of *T*. *muticellus* and the other nuclear haplotype, as well as mtDNA of *T*. *souffia*; and TEBE6 has both nuclear alleles of *T*. *muticellus*, but mtDNA of *T*. *souffia*, which implies introgression. Furthermore, one individual from the Greek Evinos River identified as *T*. *pleurobipunctatus* is in fact a hybrid of *T*. *pleurobipunctatus* and *Squalius* sp. (it possesses one nuclear allele of *T*. *pleurobipunctatus*, while another nuclear allele, as well as mtDNA belongs to *Squalius* sp. from the Evinos River, based on the BLAST search).

**Fig 5:**
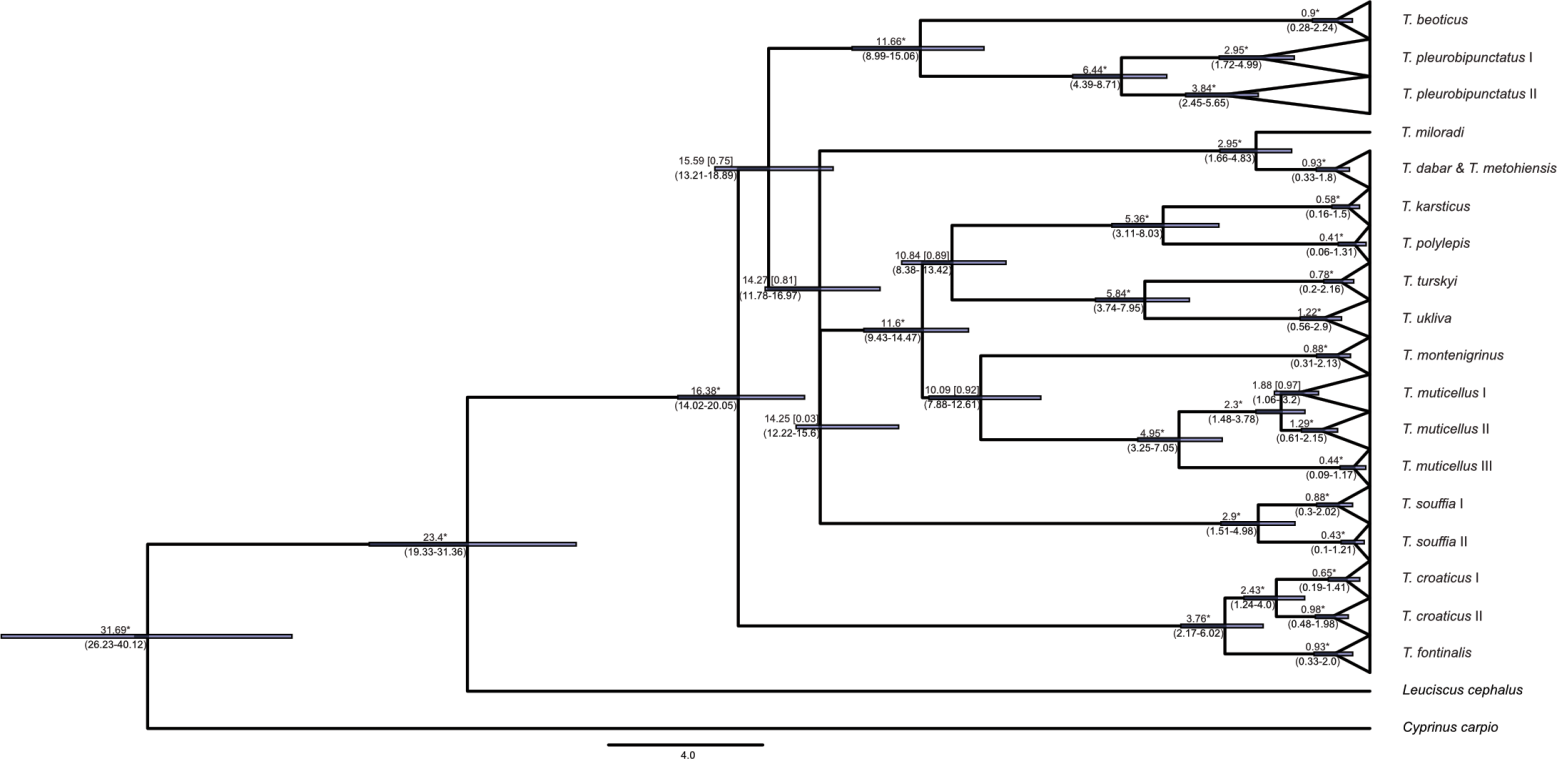
Divergence time estimations inside the genus *Telestes* based on cyt *b* sequences. Timing of the splitting events is presented by mean values and the 95% credibility range (in brackets), in million years ago. Numbers in square brackets are posterior probabilities (*-posterior probability = 1).

**Fig 6:**
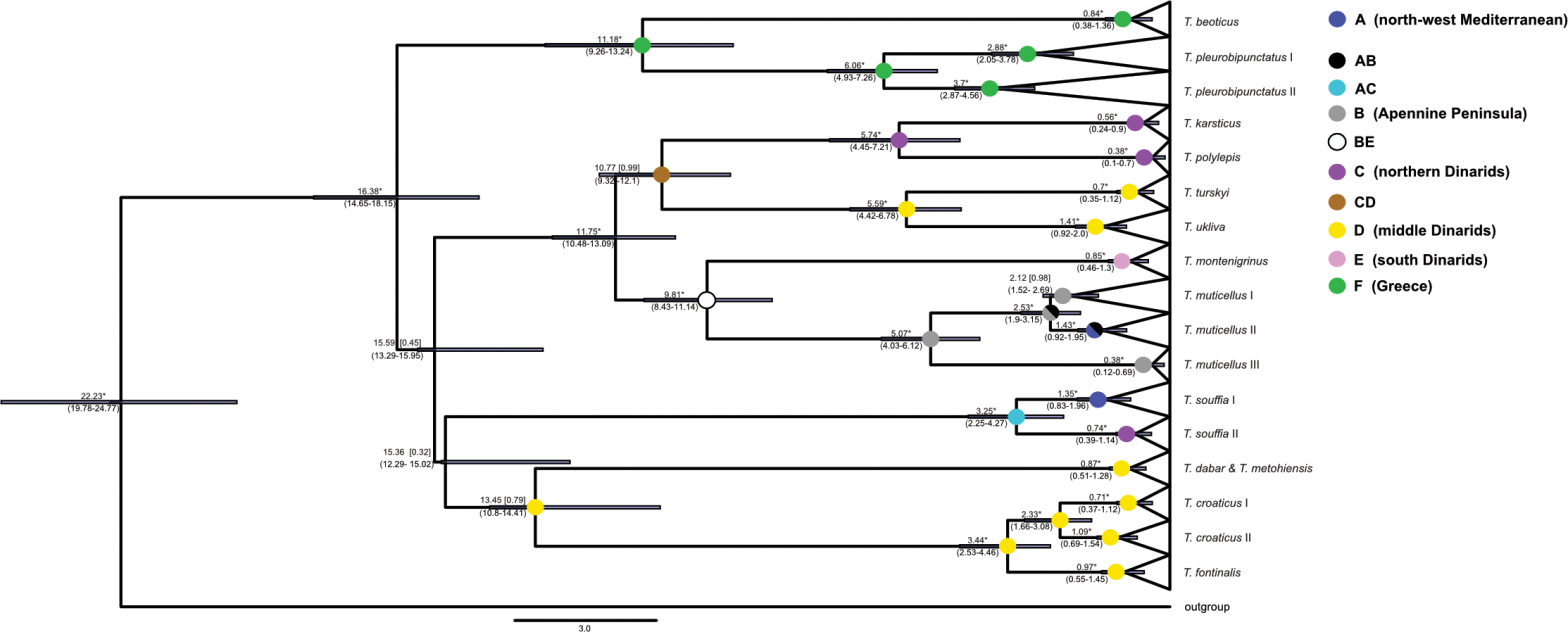
Divergence time and ancestral geographic ranges estimations inside the genus *Telestes* based on the concatenated data set (cyt *b* and RAG1). Timing of the splitting events is presented by mean values and the 95% credibility range (in brackets), in million years ago. Numbers in square brackets are posterior probabilities (*-posterior probability = 1). Ranges—A: rivers in the NW part of the Mediterranean area (Lergue, Hérault, Argens, Var, Roya, Bevera, Stafora, Po), B: Apennine Peninsula (Arno, Tiber, Volturno), C: rivers in northern Dinarids belonging to the Black Sea drainage (Soča, Gradašćica, Jevnica, Bregana, Jasenak field, Sušik, Studenac, Šmit lake), D: karstic rivers in middle Dinarids (Jadova, Ričica, Suvaja, Krbava, Čikola, Vinelić, Cetina, Gatačko field, Dabarsko filed, Konavle), E: Adriatic rivers in Monte Negro (Zeta, Morača, Cijevna), F: rivers of southern Albania and Greece (Pavllo, Kalamas, Louros, Acheloos, Evinos, Alfios, Kifissos).

Estimates for the timing of splitting events based on the cyt *b* gene are marked on the phylogenetic tree in Fig 5. Accordingly, divergence inside *Telestes* started already in the Early or in the beginning of the Middle Miocene. Several phases of the evolution can be perceived: I. ancient divergences in the Early and Middle Miocene (separation of ancestors of *T*. *croaticus*/*T*. *fontinalis*, *T*. *souffia*, *T*. *dabar/T*. *metohiensis/T*. *miloradi*, *T*. *pleurobipunctatus*/*T*. *beoticus* and the cluster containing all the remaining *Telestes* species occurred around 16.3–14.3 MYA, with divergences happening shortly one after another); II. Middle/Lower Miocene radiation inside the cluster containing the majority of *Telestes* species (origin of *T*. *muticellus*, *T*. *montenigrinus*, and the common ancestor of *T*. *karsticus*, *T*. *polylepis*, *T*. *turskyi* and *T*. *ukliva*), as well as separation of *T*. *beoticus* and *T*. *pleurobipunctatus*, all occurring around 11.6–10.1 MYA; III. Late Miocene divergences that occurred around 6.4–5.4 MYA (origin of ancestors of *T*. *karsticus*, *T*. *polylepis*, *T*. *turskyi* and *T*. *ukliva*, as well as separation of lineages inside *T*. *pleurobipunctatus*); IV. younger divergences during the Pliocene, around 5.0–2.9 MYA (separation of *T*. *miloradi* from *T*. *metohiensis*/*T*. *dabar*; *T*. *croaticus* from *T*. *fontinalis*, lineages inside *T*. *muticellus* and *T*. *souffia*). The majority of intraspecific diversity is of Late Pleistocene and Holocene origin, with the exception of species comprising more than one lineage. Very similar timing of divergence events was revealed based on the concatenated data set (Fig 6), corroborating abovementioned evolutionary phases. The only difference is the position of *T*. *metohiensis*/*T*. *dabar* lineage, which is more closely related to *T*. *croaticus* and *T*.*metohiensis* in the phylogeny based on the concatenated data set.

Ancestral range of the whole genus and higher order groupings was not determined with high probability. Nevertheless, the possibilities with the strongest support imply its ancestral range comprising areas in north and east Mediterranean region. On the other hand, ancestral ranges of younger groups (lineages and sublineages) were determined with higher support and marked on the Fig 6.

Ranges and mean values of p-distances observed in both data sets are presented in Tables 2 and 3. Mean interspecific distances, based on cyt *b* gene range between 2.4% and 10.7% and are significantly higher than intraspecific distances observed (0.2%-3.2%). The only exception is *T*. *metohiensis* and *T*. *dabar*. Mean p-distance between these two species is 0.7%. Widespread species (*Telestes pleurobipunctatus*, *T*. *souffia*, *T*. *muticellus*) and *T*. *croaticus* display higher intraspecific p-distances than recorded in the other species (3.2%, 1.2%, 1.9% and 1.2%, respectively vs. max. 0.5%). As expected, both intra- and interspecific distances were much smaller based on the RAG1 data set. High interspecific p-distances seem to be characteristic for the majority of *Telestes* species, even the ones belonging to the same phylogenetic group.

**Table 2:** Ranges and mean values (in brackets) of the p-distances among species of the genus *Telestes*, based on cyt *b* (regular letters) and RAG1 (bold letters) genes.

	FON	CRO	TUR	UKL	KAR	POL	MON	PLE	BEO	MUT	SOU	MET
*T*. *fontinalis*		**0.1–0.4** **(0.2)**	**0.8–1.0** **(0.9)**	**0.7–1.0** **(0.9)**	**0.6–0.7** **(0.7)**	**0.6–0.7** **(0.7)**	**0.8–1.0 (0.9)**	**0.7–1.3** **(1.0)**	**0.7–1.1** **(1.0)**	**0.5–1.0** **(0.7)**	**0.7–1.2** **(0.9)**	**0.4–0.7** **(0.6)**
*T*. *croaticus*	1.9–3.1 (2.4)		**0.8–1.0** **(0.9)**	**0.7–1.1** **(0.9)**	**0.5–0.7** **(0.6)**	**0.6–0.7** **(0.7)**	**0.7–1.0** **(0.9)**	**0.8–1.3** **(1.0)**	**0.9–1.1** **(1.0)**	**0.5–1.0** **(0.7)**	**0.7–1.2** **(1.0)**	**0.4–0.7** **(0.6)**
*T*. *turskyi*	9.3–10.5 (9.9)	9.4–10.2 (9.8)		**0.2–0.3** **(0.3)**	**0.4–0.5** **(0.5)**	**0.5**	**1.0**	**0.7–1.0** **(0.9)**	**0.8–0.9** **(0.9)**	**0.6–0.9** **(0.8)**	**0.8–1.1** **(0.9)**	**0.8–1.0** **(0.9)**
*T*. *ukliva*	8.3–9.6 (8.8)	8.6–9.6 (9.0)	4.1–5.1 (4.6)		**0.4**	**0.4–0.7** **(0.6)**	**0.9–1.2** **(1.1)**	**0.7–1.2** **(0.9)**	**0.7–1.0** **(0.9)**	**0.4–1.0** **(0.7)**	**0.7–1.3** **(1.0)**	**0.7–1.1** **(0.9)**
*T*. *karsticus*	8.8–10.0 (9.3)	8.8–10.2 (9.5)	7.2–7.8 (7.5)	6.3–7.3 (6.7)		**0.4**	**0.7–0.8** **(0.8)**	**0.6–1.0** **(0.7)**	**0.7–0.8** **(0.7)**	**0.2–0.7** **(0.4)**	**0.6–1.0** **(0.8)**	**0.7–0.9** **(0.8)**
*T*. *polylepis*	7.9–8.9 (8.3)	8.0–8.9 (8.4)	7.0–7.6 (7.3)	5.1–6.3 (5.6)	3.3–3.9 (3.6)		**0.6–0.7** **(0.6)**	**0.5–0.8** **(0.7)**	**0.6–0.7** **(0.6)**	**0.1–0.5** **(0.3)**	**0.6–0.9** **(0.7)**	**0.6–0.7** **(0.7)**
*T*. *montenigrinus*	8.8–10.0 (9.3)	8.6–9.6 (9.1)	7.2–7.8 (7.5)	6.3–7.5 (6.9)	6.6–7.2 (6.9)	6.2–6.6 (6.4)		**0.9–1.3** **(1.1)**	**1.0–1.1** **(1.1)**	**0.4–0.9** **(0.7)**	**0.8–1.2** **(1.0)**	**0.9–1.1** **(1.0)**
*T*. *pleurobipunctatus*	9.4–11.5 (10.3)	9.6–11.2 (10.4)	9.1–11.1 (10)	8.6–10.8 (9.7)	8.1–10.8 (9.3)	8.5–10.0 (9.2)	7.9–10.4 (9.1)		**0.2–0.6** **(0.4)**	**0.5–1.1** **(0.8)**	**0.7–1.3** **(0.9)**	**0.8–1.3** **(1.0)**
*T*. *beoticus*	9.0–10.1 (9.4)	9.2–10.1 (9.5)	10.2–10.9 (10.5)	9.2–10.4 (9.8)	8.8–9.7 (9.2)	8.1–8.6 (8.3)	8.6–9.4 (8.9)	7.4–8.6 (7.9)		**0.6–1.1** **(0.8)**	**0.7–1.1** **(0.9)**	**0.9–1.1** **(1.0)**
*T*. *muticellus*	8.2–10.2 (9.2)	8.2–10.4 (9.5)	7.9–9.4 (7.6)	6.4–7.7 (7.0)	7.3–8.6 (7.9)	6.2–7.2 (6.7)	6.4–8.4 (7.3)	9.4–11.0 (10.0)	9.1–10.6 (9.7)		**0.7–1.3** **(0.9)**	**0.7–1.0** **(0.8)**
*T*. *souffia*	8.4–9.9 (9.1)	8.7–9.8 (9.3)	8.9–9.9 (9.4)	7.9–8.7 (8.3)	7.9–8.7 (8.2)	7.1–8.0 (7.5)	7.5–8.2 (7.8)	8.0–10.3 (9.1)	8.1–9.0 (8.4)	7.5–8.9 (8.3)		**0.7–1.2** **(0.9)**
*T*. *metohiensis*	10.2–10.9 (10.5)	10.0–10.8 (10.3)	9.2–9.5 (9.4)	7.1–7.8 (7.4)	8.6–9.2 (8.9)	8.4–9.0 (8.7)	8.6–9.3 (9.0)	9.7–10.9 (10.4)	9.6–10.2 (9.8)	8.6–9.8 (9.3)	8.9–9.8 (9.4)	
*T*. *dabar*	10.3–10.9 (10.6)	10.0–10.8 (10.5)	9.4–10.0 (9.8)	7.2–8.0 (7.7)	9.0–9.6 (9.3)	8.6–9.4 (9.0)	8.9–9.6 (9.3)	9.9–10.9 (10.5)	9.6–10.3 (9.9)	9.0–10.7 (9.6)	9.2–10.2 (9.6)	0.4–1.0 (0.7)
*T*. *miloradi*	10.5–11.0 (10.7)	10.5–10.9 (10.6)	9.4–9.6 (9.5)	7.6–8.1 (7.9)	9.3–9.6 (9.4)	8.5–8.7 (8.6)	9.1–9.4 (9.3)	9.7–10.9 (10.5)	10.1–10.5 (10.2)	9.4–10.1 (9.7)	9.3–10.0 (9.6)	2.4–2.6 (2.5)

Species codes: FON–*T*. *fontinalis*; CRO–*T*. *croaticus*; TUR–*T*. *turskyi*; UKL–*T*. *ukliva*; KAR–*T*. *karsticus*; POL–*T*. *polylepis*; MON–*T*. *montenigrinus*; PLE–*T*. *pleurobipunctatus*; BEO–*T*. *beoticus*; MUT–*T*. *muticellus*; SOU–*T*. *souffia*; MET–*T*. *metohiensis*

**Table 3:** Ranges and mean values (in brackets) of the intraspecific p-distances of *Telestes* species.

	cyt *b*	RAG1
*T*. *fontinalis*	0.1–0.9 (0.4)	0.1–0.3 (0.2)
*T*. *croaticus*	0.1–2.3 (1.2)	0.1–0.2 (0.1)
*T*. *turskyi*	0.1–0.6 (0.4)	0.0
*T*. *ukliva*	0.1–0.9 (0.5)	0.2
*T*. *karsticus*	0.1–0.5 (0.3)	0.1
*T*. *polylepis*	0.1–0.4 (0.2)	0.0
*T*. *montenigrinus*	0.1–0.9 (0.5)	0.1
*T*. *pleurobipunctatus*	0.1–5.7 (3.2)	0.1–0.5 (0.2)
*T*. *beoticus*	0.1–0.7 (0.3)	0.1
*T*. *muticellus*	0.1–4.7 (1.9)	0.1–0.5 (0.3)
*T*. *souffia*	0.1 2.6 (1.2)	0.1–0.5 (0.2)
*T*. *metohiensis*	0.2–0.5 (0.3)	0.1
*T*. *dabar*	0.1–0.6 (0.4)	
*T*. *miloradi*	0.3	/

All measures of DNA polymorphisms revealed differences among species (Table 4) and high genetic variability inside the genus. Overall haplotype diversity of the cyt *b* data set was 0.99 and average number of nucleotide differences 90.42. The highest level of genetic polymorphism, based on all calculated measures, is revealed for *T*. *pleurobipunctatus* and *T*. *muticellus*. High measures are observed also in *T*. *croaticus*, *T*. *turskyi* and *T*. *ukliva*. On the other hand, *T*. *karsticus* exhibits much lower genetic diversity. In *T*. *miloradi* only one cyt *b* haplotype was observed. A similar situation was revealed based on RAG1 data set, but with differences among species less pronounced and all measures lower than when analyzing the mitochondrial marker. Higher intraspecific diversity of *T*. *muticellus*, *T*. *croaticus*, but also *T*. *souffia* was observed.

**Table 4:** Genetic polymorphism measures of *Telestes* species in cyt *b* and RAG1 genes.

Species	N	h	S	Hd	K	π	f(%)
	**cyt *b***						
***T*. *croaticus***	22	16	41	0.957	12.801	0.01131	CRO1: 18, CRO2: 14, CRO3-8: 5, CRO9: 9, CRO10-16: 5
***T*. *fontinalis***	13	7	14	0.897	3.256	0.00288	FON1: 15, FON2: 15, FON3: 8, FON4: 23, FON5: 8, FON6: 8, FON7: 23
***T*. *turskyi***	8	7	12	0.964	3.571	0.00315	TUR1: 25, TUR2-7: 13
***T*. *ukliva***	14	13	26	0.989	5.758	0.00509	UKL1: 7, UKL2: 14, UKL3-13: 7
***T*. *karsticus***	29	7	9	0.429	0.808	0.00071	KAR1: 76, KAR2-4: 3, KAR5: 7, KAR6: 3, KAR7:3
***T*. *polylepis***	5	4	4	0.9	2	0.00177	POL1: 40, POL2-4: 20
***T*. *montenigrinus***	26	10	16	0.855	4.332	0.0038	MON1: 4, MON2: 23, MON3: 19, MON4: 8, MON5: 23, MON6-10: 4
***T*. *pleurobipunctatus***	65	39	118	0.977	34.885	0.0306	PLE1: 2, PLE2: 5, PLE3: 9, PLE4: 5, PLE5: 2, PLE6: 3, PLE7: 2, PLE8: 2, PLE9: 3, PLE10: 3, PLE11-22: 2, PLE23: 3, PLE24: 2, PLE25: 2, PLE26: 3, PLE27-31: 2, PLE32: 9, PLE33: 3, PLE34: 3, PLE35: 6, PLE36-38: 2, PLE39: 5
***T*. *beoticus***	11	7	12	0.873	2.636	0.00231	BEO1: 36, BEO2:9, BEO3: 18, BEO4-7: 9
***T*. *muticellus***	52	34	96	0.976	21.857	0.01919	MUT1: 8, MUT2: 2, MUT3: 2, MUT4: 8, MUT5: 2, MUT6: 4, MUT7-9: 2, MUT10: 6, MUT11-17: 2, MUT18: 6, MUT19-21: 2, MUT22: 10, MUT23: 2, MUT24: 4, MUT25: 2, MUT26: 2, MUT27: 4, MUT29: 4, MUT30-35: 2
***T*. *souffia***	49	14	39	0.86	11.894	0.01044	SOU1: 8, SOU2: 4, SOU3: 2, SOU4: 2, SOU5: 22, SOU6: 2, SOU7: 8, SOU8: 2, SOU9: 2, SOU10: 4, SOU11: 27, SOU12: 2, SOU13: 12, SOU14:2
***T*. *metohiensis***	6	6	9	1.0	3.400	0.00299	MET1-6: 17
***T*. *dabar***	11	7	6	0.873	3.345	0.00295	DAB1: 36, DAB2-6: 9, DAB7: 18:
***T*. *miloradi***	5	1	0	0	0	0	MIL1: 100
	**RAG1**						
***T*. *croaticus***	14	7	4	0.879	1.473	0.001	rCRO1: 14, rCRO2: 29, rCRO3: 21, rCRO4-rCRO6: 7, rCRO7:14
***T*. *fontinalis***	12	3	4	0.667	1.394	0.001	rFON1:50, rFON2:17, rFON3:33
***T*. *tursky***	4	1	0	0	0	0	rTUR1: 100
***T*. *ukliva***	4	2	3	0.667	2	0.002	rUKL1: 50, rUKL2: 50
***T*. *karsticus***	28	2	1	0.071	0.071	0.000	rKAR1: 96, rKAR2: 4
***T*. *polylepis***	6	1	0	0	0	0	rPOL1: 100
***T*. *montenegrinus***	16	3	2	0.342	0.358	0.000	rMON1: 81, rMON2: 6, rMON3: 12
***T*. *pleurobipunctatus***	90	11	14	0.644	1.258	0.001	rPLE1: 57, rPLE2: 4, rPLE3: 1, rPLE4: 1, rPLE5: 4, rPLE6: 10, rPLE7: 2, rPLE8: 3, rPLE10: 11, rPLE11: 4, rPLE12: 1
***T*. *beoticus***	14	2	1	0.143	0.143	0.000	rBEO1: 93, rBEO2: 7
***T*. *muticellus***	60	16	14	0.859	2.789	0.002	rMUT1: 20, rMUT2: 2, rMUT3: 2, rMUT4: 12, rMUT5: 25, rMUT6: 2, rMUT7: 3, rMUT8: 3, rMUT9: 2, rMUT10: 3, rMUT11: 3, rMUT12: 5, rMUT13-15: 2, rMUT16: 13
***T*. *souffia***	40	9	9	0.732	1.437	0.001	rSOU1: 50, rSOU2: 5, rSOU3: 5, rSOU4: 3, rSOU5: 10, rSOU6: 5, rSOU7: 5, rSOU10: 8, rSOU11: 5
*T*. metohiensis*	12	2	2	0.303	0.606	0.001	rMET1: 83, rMET2: 17

N–number of sequences; h–number of haplotypes; S–number of polymorphic sites; Hd–haplotype diversity; K–average number of nucleotide differences; π –nucleotide diversity; f–haplotype frequency.

*Since *T*. *metohiensis* and *T*. *dabar* share the same nuclear haplotypes, they were considered a single unit for estimation of RAG1 polymorphism.

## Discussion

### Phylogenetic structure of the genus *Telestes*

Even though species of the genus *Telestes* have been included in numerous previous phylogenetic investigations (see Introduction), this is the first attempt to achieve a comprehensive depiction of its present phylogenetic structure, as well as evolutionary mechanisms that shaped it. Similar as in previous investigations, the basal lineage inside *Telestes* cannot be determined without doubt. However, the majority of the phylogenetic analyses denoted two lineages as the first ones to diverge; one comprising *T*. *souffia* (revealed by analyses based on RAG1 sequences) and the other containing *T*. *fontinalis* and *T*. *croaticus* (proposed by the majority of reconstructions of the cyt *b* data). We speculate that the reason for the discrepancy is fast and old separation event of both mentioned lineages. A long period of isolation of these ancient lineages led to their high variation compared to the remaining lineages. Further, a majority of the analyses corroborated an old origin and the distinctiveness of southern Balkan lineage containing *T*. *pleurobipunctatus* and *T*. *beoticus*. The long period of independent evolution is also corroborated by morphological features of the species belonging to the oldest lineages: *T*. *croaticus* and *T*. *fontinalis* can be distinguished from the remaining *Telestes* species by gleaming coloration (personal observation), while *T*. *pleurobipunctatus* can be differentiated from the remaining species by having pharyngeal teeth in one row only [29], [3].

The remaining phylogenetic lineages form a common cluster, in which the phylogenetic relationships do not always follow a geographic pattern, e.g. *T*. *croaticus* and *T*. *fontinalis* are very distant from the remaining species distributed in northern Dinarids; *T*. *montenigrinus* seems to be related to *T*. *muticellus*, even though their recent distribution ranges are apart. This investigation, furthermore, rejected the presumption of a closer relatedness of *T*. *souffia*, *T*. *muticellus* and *T*. *pleurobipunctatus*, which were sometimes considered to form a single complex [3].

Recent relationships and intraspecific structuring was better resolved with mitochondrial marker, due to its faster mutation rate, whereas nuclear phylogeny seems to be adequate for describing speciation. Significant intraspecific structuring and the existence of more than one lineage are present in *T*. *souffia*, *T*. *muticellus*, *T*. *croaticus* and *T*. *pleurobipunctatus*. Divergence of two *T*. *souffia* lineages is of Pliocene origin and is concordant with findings of previous authors [30], [9] with one lineage distributed in France (rivers Argens, Hérault, Lergue and Var as the type locality) and a second lineage inhabiting localities in the Danube basin, as well as the Soča River. Two *T*. *souffia* lineages were denoted also by [5], whereas [4] recognized four distinct lineages inside *T*. *souffia*, one of which is concordant with our second lineage. Previous reports regarding *T*. *muticellus* were contradictory (e.g. [8] described low genetic diversity of that species, whereas [6] presented five distinct haplotype groups), and sometimes not reliable due to the inclusion of *T*. *souffia* samples (e.g. in the investigation of [6]). Based on our results, *T*. *muticellus* comprises at least three highly distinct lineages: I. Volturno population, whose distinction was already observed by [31], II. populations from the northern part of *T*. *muticellus* distribution range, III. Arno and Tiber populations. Two lineages of *T*. *croaticus* diverged in the late Pliocene or early Pleistocene. However, haplotypes belonging to both lineages are present in the Jadova R., implying their secondary contact. Exceptionally pronounced structuring is also present inside *T*. *pleurobipunctatus*.

In order to investigate more detail intra- and interpopulational, as well as within lineages structure and gene flow, further investigations are advisable that should focus on a certain lineage, but include more genetic markers.

### Evolutionary history scenario

**Fig 7:**
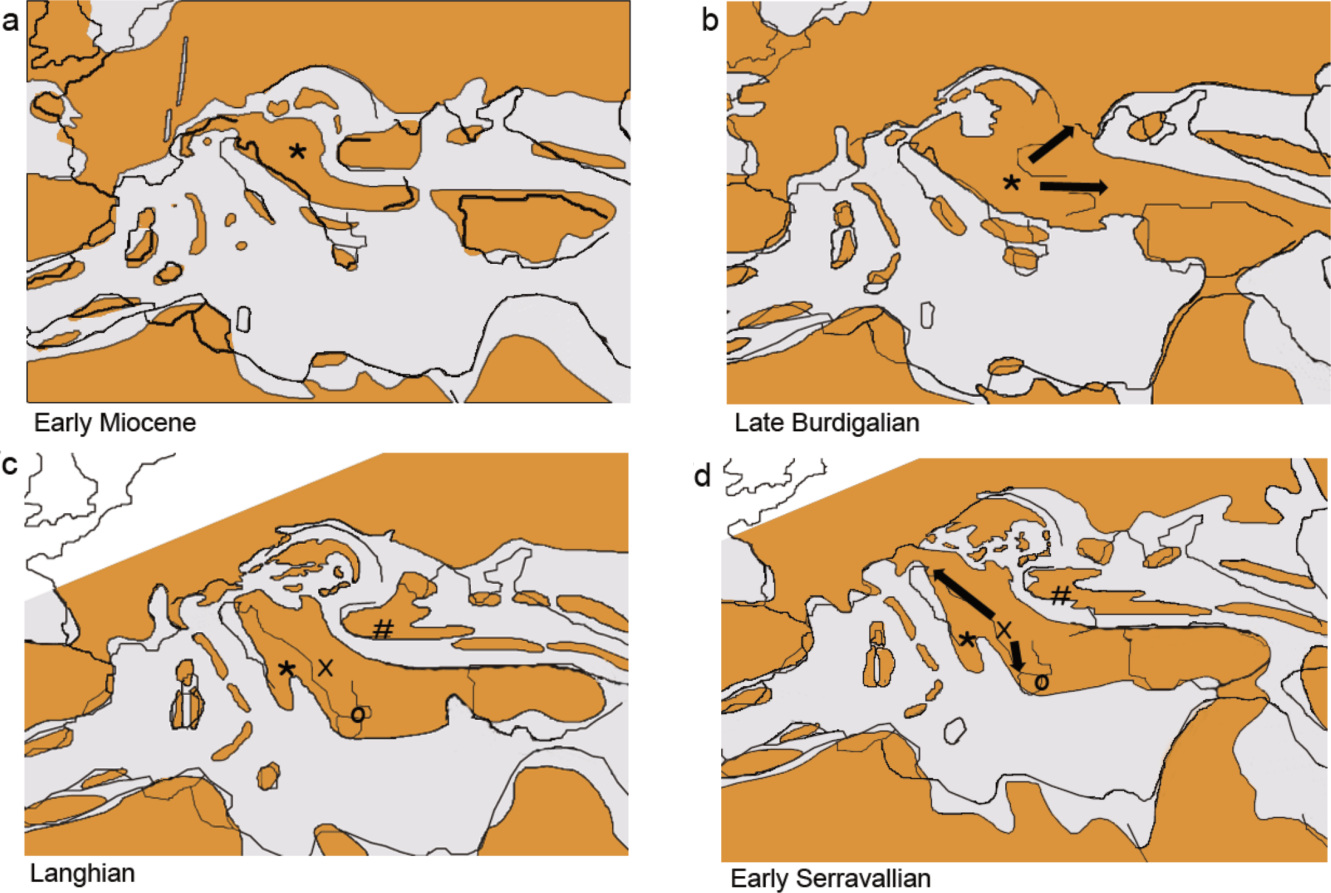
Colonization routes of the *Telestes* lineages, based on the proposed evolutionary scenario. *—*Telestes* ancestor in 7a and 7b, ancestor of *T*.*croaticus/T*. *fontinalis* in 7c and 7d; #—ancestor of *T*. *souffia*; x–ancestor of the species cluster (*T*. *metohiensis/T*. *dabar/T*. *miloradi/T*. *muticellus/T*. *montenigrinus/T*. *ukliva/T*. *turskyi/T*. *karsticus/T*. *polylepis*); o–ancestor of *T*. *pleurobipunctatus/T*. *beoticus*.

The proposed evolutionary scenario (Fig 7) relates to estimates based on both cyt *b* gene and nuclear data set, that resulted in similar estimation of timing of divergence events and known data on the historical biogeography of Europe ([32], [33], [34], [35]). Reconstruction of ancestral ranges, even though not reliable for older events, is also concordant with the proposed scenario, as explained below.

In the first stage of the Miocene, southern Europe was an archipelago, with an ancestor of *Telestes* probably persisting on one of the islands (Fig 7A), which it might have reached from the European mainland or Anatolia during earlier landmass connections. However, by the end of the Burdigalian (16 MYA), some of the islands became joint into a single landmass, forming a continental bridge between western Europe and Anatolia [33] and providing an opportunity for colonization of eastern Balkan region (concordant with the divergence of *T*. *pleurobipunctatus*/*T*. *beoticus* lineage) (Fig 7B). In the same period the northern part of the Balkan was connected with central Europe and the Dinarids were not formed yet, explaining the connection of *T*. *souffia* with the Mediterranean species. Phylogenetic reconstructions and divergence time estimations imply the hypothesis that already in the Burdigalian, when connections between central Europe, Balkan and Anatolia were possible [33], the ancestor of *T*. *croaticus* and *T*. *fontinalis* was isolated. In the Langhian stage (15.9–13.8 MYA), following the Middle Miocene transgression, the majority of central and southern Balkan became separated from the European landmass, with several land fragments also persisting in the Parathetys [33]. The ancestor of *T*. *croaticus* and *T*. *fontinali*s did not come into a secondary contact with the remaining *Telestes* lineages, leading to the clear distinctiveness between those two species and the remaining ones, even though some of them (e.g. *T*. *polylepis* and *T*. *karsticus*) are presently located in their immediate geographic proximity. By the Langhian stage (Fig 7C) *T*. *souffia* was also isolated from the remaining lineages, and its further evolutionary course was determined by the biogeography of the Black Sea watershed and central Europe. Nevertheless, on the already mentioned southern island the diversification of *Telestes* continued and consequently led to the high species richness. During the Langhian phase the ancestor of Herzegovinian and southern Dalmatian *T*. *metohiensis*, *T*. *dabar* and *T*. *miloradi* became isolated from the remaining species belonging to already mentioned species cluster. The divergence of *T*. *muticellus*, distributed on the Apennine peninsula can be dated to the Serravallian stage (12.6–7.8 MYA, Fig 7D). The colonization of the Apennine peninsula might be provoked by the closing of the Slovenian corridor. Furthermore, communications between the Black Sea and the Adriatic watershed also existed in that period, at least until the early Tortonian (11.6 MYA) when lineages of *T*. *karsticus*/*T*. *polylepis* and *T*. *turskyi*/*T*. *ukliva* diverged. Separation of species inside the mentioned lineages occurred in the Messinian stage (7.2–5.3 MYA), and is probably connected with Dinaric Lake System [34], [35] and/or intense tectonic activity. The Pliocene epoch had a final impact on *Telestes* diversity, because divergence of several species (*T*. *metohiensis*, *T*. *miloradi*) or lineages within *T*. *muticellus*, *T*. *souffia* and *T*. *pleurobipunctatus* can be dated back to that time.

Our estimates for early divergences inside this genus are older than proposed by previous authors [7], [1], [8], [4], which is probably a result of molecular clock calibration and smaller sample sizes included in earlier investigations. In disagreement to previous investigators [1], [4], our results do not point out the Messinian Salinity Crisis (MSC) as critical point for the divergence of Mediterranean *Telestes* species, supporting findings of [2] for the Leuciscinae, but also for Adriatic spined loaches (genus *Cobitis*; [36]). It is also clear that the separation of *T*. *souffia* and *T*. *muticellus* did not happen simultaneously, as previously proposed [5], but evolutionary history of *Telestes* can rather be described as containing sequences of gradually occurring events intermittent with longer periods that did not leave a trace in recent genetic structure.

Even though widely observed inside Leuciscinae, hybridization did not play an important role in the evolutionary history of *Telestes*, although it was sporadically noticed. Previous investigations [37], [5] revealed recent gene flow between *T*. *souffia* and *T*. *muticellus*, which was explained as hybridization by [4]. Since we have investigated both nuclear and mitochondrial DNA, we are able to conclude that interspecific connections include both nuclear hybridization and introgression of mtDNA, but are very restricted based on our sampling.

Contrary to the opinion of [1] that relationships of *Telestes* species mostly follow the geographic pattern, it seems that the complex geological history of southern Europe led to complicated biogeographical pattern with genetically distinct species distributed close to each other, most likely a consequence of multiple colonization and crossing events between rivers and water basins. For example, in the Adriatic watershed in Croatia and Bosnia and Herzegovina species belonging to three lineages are present, implying three colonization events of this area by different lineages and in different time periods, explaining the high diversity observed there.

### Molecular diversity of *Telestes* species

The distribution range of *Telestes* comprises the Mediterranean and central European area. Inside its range there are smaller regions with exceptionally high *Telestes* diversity both, at the species and genetic levels. The Eastern Adriatic coast is the species’ ‘hot spot’, but also contains high levels of genetic diversity, especially in the region of central Dalmatia (Krka and Cetina River drainages). Even though the number of recognized species is smaller in Greece, it contains the highest genetic diversity of *Telestes* at all.

Elevated levels of genetic polymorphisms based on the mitochondrial marker (Hd>0.95) were found in *T*. *pleurobipunctatus*, *T*. *muticellus* and *T*. *croaticus*, three species with pronounced intraspecific structuring; but also inside *T*. *turskyi* and *T*. *ukliva* (Table 4). The latter two species have smaller distribution ranges and their high genetic diversity is a probable consequence of their old origin and unconstrained evolutionary history, due to the absence of glaciations in their distribution ranges that might have provoked bottleneck events. The main difference in the genetic polymorphism measures between these two groups of species is in the values of nucleotide diversity and the average number of nucleotide differences that are significantly higher in well-structured species (34.9 in *T*. *pleurobipunctatus*, 21.9 in *T*. *muticellus* comparing to maximum of 5.8 in species with homogenous structure). Likewise, *T*. *souffia*, another species comprising more than one genetically distinct unit, also contains high average number of nucleotide differences and high nucleotide diversity, although its haplotype diversity is smaller. The nuclear RAG1 gene also pointed out *T*. *croaticus*, *T*. *muticellus*, *T*. *souffia*, *T*. *ukliva*, *T*. *fontinalis* and *T*. *pleurobipunctatus* as genetically highly diverse. Very high values of nucleotide diversities and average number of nucleotide differences contained inside *T*. *pleurobipunctatus*, *T*. *muticellus* and *T*. *souffia*, are not characteristic for a single species and imply the possibility that they represent cases of species complexes, which is concordant also with other results and some previous reports. Similarly as obtained in this investigation for cyt *b* gene, inadvertent inclusion of cryptic species in samples presumably belonging to a single species has been considered as one of the most powerful causes of bias in haplotype and nucleotide diversity estimations based on mitochondrial cytochrome *c* oxidase sequence data [38].
